# Integrative analysis of large scale expression profiles reveals core transcriptional response and coordination between multiple cellular processes in a cyanobacterium

**DOI:** 10.1186/1752-0509-4-105

**Published:** 2010-08-02

**Authors:** Abhay K Singh, Thanura Elvitigala, Jeffrey C Cameron, Bijoy K Ghosh, Maitrayee Bhattacharyya-Pakrasi, Himadri B Pakrasi

**Affiliations:** 1 Department of Biology, Washington University, St. Louis, MO 63130, USA; 2 Department of Electrical and Systems Engineering, Washington University, St. Louis, MO 63130, USA; 3 Department of Mathematics and Statistics, Texas Tech University, Lubbock, TX 79409, USA

## Abstract

**Background:**

Cyanobacteria are the only known prokaryotes capable of oxygenic photosynthesis. They play significant roles in global biogeochemical cycles and carbon sequestration, and have recently been recognized as potential vehicles for production of renewable biofuels. *Synechocystis *sp. PCC 6803 has been extensively used as a model organism for cyanobacterial studies. DNA microarray studies in *Synechocystis *have shown varying degrees of transcriptome reprogramming under altered environmental conditions. However, it is not clear from published work how transcriptome reprogramming affects pre-existing networks of fine-tuned cellular processes.

**Results:**

We have integrated 163 transcriptome data sets generated in response to numerous environmental and genetic perturbations in *Synechocystis*. Our analyses show that a large number of genes, defined as the core transcriptional response (CTR), are commonly regulated under most perturbations. The CTR contains nearly 12% of *Synechocystis *genes found on its chromosome. The majority of genes in the CTR are involved in photosynthesis, translation, energy metabolism and stress protection. Our results indicate that a large number of differentially regulated genes identified in most reported studies in *Synechocystis *under different perturbations are associated with the general stress response. We also find that a majority of genes in the CTR are coregulated with 25 regulatory genes. Some of these regulatory genes have been implicated in cellular responses to oxidative stress, suggesting that reactive oxygen species are involved in the regulation of the CTR. A Bayesian network, based on the regulation of various KEGG pathways determined from the expression patterns of their associated genes, has revealed new insights into the coordination between different cellular processes.

**Conclusion:**

We provide here the first integrative analysis of transcriptome data sets generated in a cyanobacterium. This compilation of data sets is a valuable resource to researchers for all cyanobacterial gene expression related queries. Importantly, our analysis provides a global description of transcriptional reprogramming under different perturbations and a basic framework to understand the strategies of cellular adaptations in *Synechocystis*.

## Background

Genomic scale measurements of cellular components such as RNA, protein, and metabolites have been instrumental in unraveling the complexity of cellular functions in *E. coli*, yeast and other model organisms [1-9]. One group of organisms, namely cyanobacteria, lag behind in the use of these technologies, despite its evolutionary, ecological, environmental and biotechnological importance. Cyanobacteria are the only known prokaryotes capable of oxygenic photosynthesis and they play a significant role in global biogeochemical cycles [10]. It is estimated that cyanobacteria may be responsible for more than half the total primary production essential for sustaining life on Earth [11]. These organisms are credited with the transition of the Earth's atmosphere from an anaerobic state to the aerobic condition, the evolution of planetary primary production, and as being the progenitor of chloroplasts in higher plants [12-14].

A systems level understanding of changes in cellular components, and coordination among elaborate networks of fine-tuned cellular processes in cyanobacteria is of the utmost importance for many reasons. For example, cyanobacteria have recently attracted significant interest due to its crucial role in carbon capture, and its ability to produce renewable carbonneutral biofuels. Additionally, cyanobacteria are good model systems to understand the impact of changing conditions on an organism's physiology. This is because cyanobacteria have successfully survived broad changes in environment including temperature, pH, nutrient availability, redox, CO_2 _and O_2 _throughout evolution [15]. Similarly, the exquisite dependence of these organisms on sunlight for the generation of energy for their entire metabolism necessitates constant modifications of the cellular machinery, such that light energy can be efficiently harvested under natural conditions. These characteristics have enabled cyanobacteria to acquire diverse arrays of physiological characteristics which are necessary for survival under changing environmental conditions. Identification of key factors and understanding their role in the acquisition of phenotypic states under changing environmental conditions has been an active area of research in cyanobacteria for many years. Among cyanobacterial strains, *Synechocystis *sp. PCC 6803 (hereafter *Synechocystis*) has been extensively used as an experimental organism for physiological, biochemical and molecular studies. The availability of the *Synechocystis *genome sequence, the first cyanobacterium and the third prokaryote to be sequenced [16], has allowed a spurt of large scale studies on measurements of RNA and proteins in this model organism.

Numerous gene expression data sets for *Synechocystis *under diverse environmental and genetic conditions (see additional file 1) have accumulated in the literature and public databases. However, these transcriptome studies in *Synechocystis *have not led to a full understanding of the interactions between cellular processes required to maintain a steady internal state (homeostasis) for optimal function and growth. A major reason for this is related to the use of a random cut-off approach in most studies to identify differentially regulated genes. As a result, genes with low transcript abundance or showing small changes in transcripts levels have not been identified in most of these studies. The importance of the identification of such genes is being increasingly recognized, because organisms have stronger constraints on their expression patterns due to their involvement in crucial cellular functions [17]. Additionally, recent studies have shown that transcriptome reprogramming plays a central role in the acquisition of a phenotypic state under specific perturbations [1,8,18-20]. Thus, identification of all differentially regulated genes in *Synechocystis*, regardless of the amplitude of changes in transcript levels, is very important to understand cellular strategies when cells are perturbed.

Significant insights on integrated cellular response from omics data sets, especially transcriptomics, have been obtained by the integration of data sets generated under diverse genetic and environmental perturbations [4-7,9,21-23]. Such an approach offers many advantages over studies involving a single perturbation. For example, integration of data sets readily allows identification of genes with low transcript abundance or small changes in transcripts levels. Additionally, stress-specific and general responses can also be identified by the integration of data sets. The availability of large data sets also makes it possible to derive a realistic, systems level network based on the regulation of metabolic pathways identified from the expression patterns of their associated genes. Such a network provides the framework to identify coordination and interactions between various cellular processes under different perturbations, and is key to an overall understanding of how various cellular functions coordinate to develop appropriate adaptation strategies. In this study, we have integrated transcriptomic data sets obtained from *Synechocystis *under 163 different environmental and genetic perturbations. This has led to the identification of genes differentially regulated in response to most perturbations. Furthermore, availability of large data sets enabled us to generate a Bayesian network based on KEGG pathways which has provided insights into the interactions among cellular pathways.

## Results and Discussion

### Analysis of the *Synechocystis *transcriptome under multiple perturbations

We initiated an examination of adaptation strategies in *Synechocystis *to various stress conditions by integrating transcriptome data sets from 163 environmental and genetic perturbations. These data sets were obtained from various public sources (see Methods for details). The complete description of experimental conditions, source(s) of transcriptome data sets and relevant references are provided in the additional file 1. Integration of transcriptome data sets obtained using differing microarray platforms/laboratories was challenging from a data analysis perspective. In addition, physiological conditions of *Synechocystis *cells in several of the studies under normal conditions as well as after the exposure to perturbations were not documented. This further complicated our analysis. We reasoned that if the adaptation strategies employed by *Synechocystis *under different perturbations are robust, it should be possible to identify them despite the above-mentioned technical and biological challenges. Our analysis framework included normalization of raw data using a robust version of LOWESS [24], and the identification of differentially regulated genes using a statistical significance test on log-transformed ratios of changes in gene expression between cells under control and perturbed conditions. The amplitude of changes in transcript levels was ignored, largely because such changes are subject to the magnitude of the perturbations both in intensity and duration. For example, expression of heat shock proteins (HSPs) responds immediately to changes in increased growth temperature, whereas, for the same genes, the response to changes in nutritional deficiency may be slower. In addition, the unknown physiological conditions of cells make selection of differentially regulated genes based solely on the amplitude of transcript level meaningless. We discretized the regulation of differentially expressed genes into three levels; upregulated (+1), downregulated (-1) and not regulated (0), such that the final conclusion is not biased by the contribution of individual experiments. This step was performed for each experiment separately to ensure that the analysis was independent of technical variations. We also removed genes present on the six plasmids in *Synechocystis *from further analysis because data on the regulation of plasmid encoded genes has been mostly studied only in our lab [25]. All 163 transcriptome data sets were considered for pathway-level analyses whereas gene-level analyses were based on 68 data sets due to the availability of sufficient number of replicates allowing statistical significance test of the data sets.

**Figure 1:**
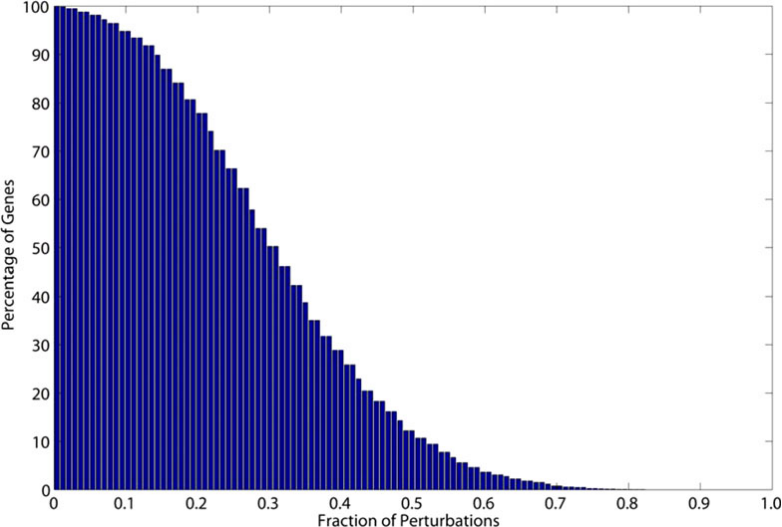
**Differential regulation of *Synechocystis *genes across multiple perturbations**. Differential regulation of genes was assessed by using a statistical significance test. Only 68 data sets generated under various environmental and genetic perturbations had sufficient number of replicates to be considered for the determination of such differential regulation of genes. All but 5 genes present on chromosome (3259/3264) were identified as differentially regulated in at least one experimental data set. The complete data sets with their respective fold changes are provided in the additional file 2.

We could identify all but five genes (3259/3264) present on the *Synechocystis *chromosome that show differential regulation in at least one study (Fig. 1; additional files 2 and 3). As expected, integration of data sets readily allowed a separation of stress-specific genes from general-stress response. The comparative analysis of differentially regulated genes shows that a large number of genes respond in a stress-specific manner, showing differential expression that was limited to one or a group of stress conditions. These genes may play a direct role under a given perturbation or under certain conditions that have similar effects on cell physiology. It is also quite possible that a fraction of these stress-specific genes are identified purely based on the noise and/or technical issues associated with the individual study. Nonetheless, we expect that compilation of the transcriptome data sets in the present study will be a useful resource towards understanding the functional roles of gene products in *Synechocystis *as well as in the functional annotation of hypothetical and unknown genes. We encourage readers to explore the regulation of genes provided in the additional file 2. We have found the transcriptome data sets useful in many ways. For example, one can easily identify genes or cellular processes that are specifically regulated in response to a certain stress. The entire data sets are available online at http://www.fibr.wustl.edu/synecho6803_database/.

Further analysis of the *Synechocystis *transcriptome shows that a large number of genes were differentially regulated under a majority of perturbations. We could identify approximately 12% (399) of *Synechocystis *chromosomal genes that show differential regulation in more than 50% of transcriptome data sets (additional file 4). Since these genes exhibit consistent differential regulation in the majority of perturbations, we have designated these genes collectively as the 'core transcriptional response (CTR)' in *Synechocystis*. Expression of the CTR under diverse conditions attests to its significant role in adaptation strategies. In addition, regulation of the CTR is much more significant when only data sets obtained under environmental perturbations are examined. The regulation of the CTR in at least half of the perturbations suggests that changes in transcript levels of these genes may not be directly related to the primary stress but are secondary effects. In this scenario, it is likely that a combination of stress-specific and the CTR genes highlight the uniqueness of each transcriptional program utilized to choose a suitable phenotypic state under a given perturbation. Of the 399 genes, the majority are involved in the photosynthetic process (79 genes), transcription and translation (30 genes), energy metabolism (22 genes), HSPs (10 genes), and nitrogen metabolism (8 genes). The remaining genes in the CTR encode proteins involved in other processes (79 genes) and for unknown functions (165 genes). Below, we provide a short description of the major processes present in the CTR and describe the impact of their regulation on the adaptation strategies during various perturbations.

#### Photosynthesis

All organisms need chemical energy for metabolic activities. For *Synechocystis*, photosynthesis is the sole source to generate chemical energy under photoautotrophic conditions. For this reason, there is a tight connection between the activity of the photosynthetic process and other principal metabolic pathways. It is not surprising therefore that photosynthesis was one of the major cellular functions represented in the CTR. More than 60% of all photosynthesis genes in *Synechocystis *were present in the CTR and they were downregulated across most perturbations. Of the various functional complexes, genes involved in ATP synthase (all genes), CO_2 _fixation, phycobilisome, PSI and PSII were most affected under different perturbations. There were fewer genes encoding NAD dehydrogenase in the CTR and their regulation did not follow the pattern similar to the regulation of other photosynthetic complexes. In addition to its role in providing energy for cellular metabolism, the intermediates of photosynthetic electron transport chain have been suggested to monitor the overall physiological conditions through redox active intermediates [26,27]. It has been suggested that the redox state of plastoquinone (PQ), an electron carrier that transfers electrons from PSII to cytochrome b6f, is involved in the regulation of photosynthetic genes among other genes. In this scheme, oxidized PQ controls PSII genes whereas reduced PQ controls PSI genes. Our analysis shows that the majority of perturbations led to downregulation of both PSII and PSI genes, suggesting against the involvement of the redox state of PQ as the principal regulator of photosynthesis genes. Regardless of the factors involved in photosynthesis gene expression, it is evident from our analysis that downregulation of photosynthetic activity is key to acclimation under a majority of perturbations. Our analysis additionally shows that the regulation of photosynthesis genes in *Synechocystis *is associated with the general stress response.

#### Energy metabolism

*Synechocystis *contains active glycolysis/gluconeogenesis pathways. In most cases, reduced carbon generated from photosynthesis enters the glycolytic pathway and is subsequently utilized in many biosynthetic pathways. In some conditions, reduced carbon is stored in the form of glycogen which can be utilized later to provide energy, and as a carbon source for many biosynthetic pathways [28]. Our analysis shows that energy metabolism is another functional category for which a number of genes were present in the CTR. Most of these genes were also downregulated across the different experimental conditions. Thus, it is clear from our analysis that most perturbations lead to downregulation of energy production through the two major routes, photosynthesis and energy metabolism, in *Synechocystis*. These results are in accordance with studies from other organisms, where shutting down major energy producing pathways have been suggested to be important in acclimation under stress conditions.

#### Translation

Translation is another process for which several genes were present in the CTR, and in general, they were downregulated. Downregulation of ribosomal genes suggests that most perturbations in *Synechocystis *result in the transient translational/growth arrest similar to what has been suggested for several other organisms [4,18,21,22]. It is noteworthy that expression of ribosomal genes in *Synechocystis *correlates with regulation of energy producing pathways (photosynthesis and energy metabolism). Thus, it can be suggested that regulation of genes involved in photosynthesis, energy metabolism and translation are controlled in anticipation of changes in growth under perturbations.

#### Stress proteins

Genes encoding a number of proteins with known functions in cellular protection and repair including HSPs, proteases, sigma factors, and antioxidant proteins were present in the CTR and they were upregulated across perturbations. Upregulation of genes encoding stress proteins are suggestive of two intertwined attributes; damage to proteins and increased production of reactive oxygen species (ROS) due to cellular redox imbalance caused by perturbation. The maintenance of redox balance is particularly challenging for oxygenic photosynthetic organisms like *Synechocystis *because photosynthesis inherently produces strong oxidants and reductants. Additionally, multiple redox active intermediates are used as electron carriers in the photosynthetic electron transport chain. Thus, any disturbance in the photosynthetic electron transport chain and/or activity of enzymes involved in photosynthesis significantly influences the redox poise leading to an increased production of ROS.

#### Regulatory genes

The CTR contains six regulatory and sensory genes. Four of these genes (*sll1742, slr1738, slr1285 *and *sll1392*) were upregulated while two (*sll1555 *and *sll1291*) were downregulated. Interestingly, known genes under the control of these regulatory proteins were also part of the CTR suggesting that the regulatory and their target genes may be co-regulated. For example, the *slr1285 *(*hik34*) gene has been suggested to be involved in expression of HSP genes [29], and as described in the previous section, several HSP genes are part of the CTR. Both *hik34 *and HSP genes were upregulated in most perturbations. Similarly, the *sll1392 *gene has been named *pfsR*, an acronym for photosynthesis, Fe homeostasis, and stress response [30]. Although, little is known about genes under the control of PfsR, we find that numerous genes belonging to photosynthesis, Fe homeostasis, and stress response are part of the CTR. A third gene, *sll1291 *(*rre12*), is part of a gene cluster involved in regulating chemotaxis. Chemotaxis is a general phenomenon found in bacteria, and is required for optimal growth under unfavorable conditions. In addition, we find that regulation of the *hik42 *gene, a hybrid sensor and regulator, is conserved across perturbations suggesting an important role for its product in the development of appropriate adaptation strategies. Finally, the *slr1738 *gene encoding PerR, was also present in the CTR. PerR is a transcriptional regulator belonging to the Fur family of regulators that negatively controls a set of genes responding specifically to oxidative stress [31-33].

#### Genes with unknown functions

Nearly 40% of the genes in the CTR encode proteins with unknown functions. Some of these genes have orthologs in at least another cyanobacterial strain while some are only present in *Synechocystis*. These specific *Synechocystis *genes may impart their unique signature features necessary for survival in the natural habitat. Identification of these unknown genes in the CTR provides a strong foundation to determine their functional roles in the development of adaptation strategies under perturbations. For example, we find that a gene cluster, *sll1783-sll1785*, is consistently regulated across perturbations. A literature survey shows that the *sll1785 *gene has recently been implicated in the transport of copper [34]. We hope that the identification of the commonly regulated unknown genes will spur research work to identify their crucial role(s) in cyanobacterial physiology.

#### Other genes

In addition to the above described functional groups, genes involved in other cellular processes were also present in the CTR (additional file 4). However, the numbers of genes regulated in these pathways were not as large when compared to the functional groups described in the previous sections. A primary reason for the lesser number of genes could be related to our observation that the same gene(s) in a given pathway were not always regulated across various experimental conditions. It remains to be seen if these pathways are controlled by differential regulation of different set of genes specific to certain conditions.

### Regulation of the CTR

We next asked whether transcriptome data sets can provide insights into the mechanism regulating the CTR. We considered two possibilities: (i) a single regulatory system responding to an internal signal commonly generated under most perturbations, or (ii) a complex set of regulatory network systems responding to either internal or external signals. In the latter case, change(s) in the components and/or functional states of a regulatory system under a specific condition leads to an overall modulation of the network. This in turn triggers the regulation of the CTR. An indication of the potential mechanism(s) comes from regulation of genes involved in photosynthesis, energy metabolism, nutrient assimilation and translation described in previous sections. A majority of these genes showed similar pattern(s) of regulation, which is in agreement with the suggestion that photosynthesis is tightly connected with other metabolic pathways. Thus, it can be suggested that regulation of the CTR under perturbation is linked to the activity of the photosynthetic electron transport chain. Such a hypothesis has been considered previously. It has been suggested that the redox state of PQ is involved in communicating environmental signals to regulatory circuits such as the one that controls the CTR [26,27,35,36]. However, our analysis shows that the regulation of photosystem genes is not dependent on the redox state of PQ. This finding is in accordance with a previous study which showed that treatment of *Synechocystis *with saturating amount of DCMU and DBMIB led to downregulation of photosynthetic genes [37]. We propose that regulation of the CTR is controlled by ROS. Evidence for the involvement of ROS comes from the identification of numerous upregulated genes involved in protection against ROS including superoxide dismutase, *aphC *(encoding a type-2 peroxiredoxin) and *perR*. Upregulation of superoxide dismutase provides support to our argument that most perturbations lead to increased production of hydrogen peroxide in the cell. Additionally, expression of *aphC *and *perR *genes, both expressed from the same divergent promoter in *Synechocystis*, is known to be responsive to hydrogen peroxide [31,32]. Our analysis also provides evidence for the involvement of multiple regulatory and sensory proteins in regulation of the CTR in *Synechocystis*. Evidence for this comes from the presence of several regulatory genes and their target genes in the CTR (additional file 4). These results together suggest that multiple regulatory network systems sense internal signal(s) in the form of ROS produced under various perturbations to regulate the CTR. We note that this conclusion is based entirely on the expression patterns of genes. Further experimentation based on these findings will be necessary to establish a role of ROS in the regulation of the CTR.

**Figure 2:**
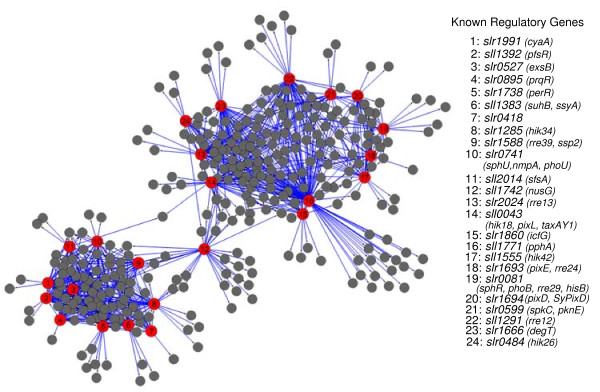
**Coregulation of regulatory and CTR genes**. A correlation matrix was generated for the 146 regulatory genes and 399 genes present in the CTR by using the Hamming distance. Based on previous experimental results, we used 60% agreement as a cut-off to identify coregulation. Using this criterion, we could identify 25 regulatory genes (red solid dots) that were coregulated with 85% of the genes present in the CTR (black solid dots). The relevant similarity measurement values are provided in the additional file 5.

Large-scale transcriptomic data sets have been useful in identifying groups of genes showing similar patterns of expression. Such analyses have been used to predict functions of unknown genes based on coregulation with functionally known genes and for the identification of target genes of transcriptional regulators. The latter is based on the assumption that regulatory factors are themselves transcriptionally regulated such that their expression patterns are strongly correlated with target genes. To identify whether genes in the CTR are coregulated with any of the regulatory genes in *Synechocystis*, we developed a correlation matrix for 146 regulatory genes. We identified genes as being coregulated with a given regulatory gene, if their expression profiles correlate in at least 60% of the experiments. The use of this criterion is based on the expression profiles of two genes, *perR *(a regulatory gene) and *aphC *gene, divergently transcribed from a common promoter. Transcription of these two genes is negatively controlled by PerR, which binds to a single PerR site present in the promoters of these genes. Inactivation of PerR leads to simultaneous expression of both genes [31,32]. Our analysis shows that expression profiles of these two genes were similar in 61% of the experiments. We found that expression patterns of 25 regulatory genes could be correlated with more than 85% of genes found in the CTR (Fig. 2). As can be expected, expression patterns of some genes were found to be correlated with multiple regulatory genes while others showed coregulation with a single regulatory gene (additional file 5). Interestingly, the expression patterns of several unknown genes were found to be correlated with multiple regulatory genes.

A correlation between expression patterns for regulatory and target genes provides the first step towards a full understanding about the existence of regulatory networks. This is especially true for *Synechocystis *where little is known about the existence of such networks. To begin with, we looked for the previously reported regulatory factors and their target genes and whether our analysis was able to capture them. We find that several HSP genes are coregulated with the *hik34 *gene (Fig. 2; additional file 5). As previously mentioned, Hik34 has been suggested to be involved in the regulation of HSP genes in *Synechocystis *[29]. These results indicate that identification of coregulated genes could provide useful information on regulatory networks. Next, we attempted to determine whether target genes contain conserved cis-elements involved in the binding of transcriptional regulators. Unfortunately, not many cis-elements specific to transcriptional regulators have been identified in *Synechocystis*. Of the regulatory genes selected, only the binding site of PerR is known. A 15-bp consensus sequence (TTATAATnATTATAA) is known to be required for the binding of PerR in *Bacillus subtilis *[38]. A similar binding site has been suggested in *Synechocystis *[32]. Our analysis shows that a total of 53 genes were coregulated with the *perR *gene. Interestingly, expression patterns of some genes were strongly correlated with the *perR *gene; even better than the correlation with the *aphC *gene. A survey of the PerR binding site in the upstream regions of all 53 coregulated genes showed that very few genes contain such a repeat. In fact, we were unable to find a canonical binding site for PerR in the upstream region of genes which showed stronger correlation to PerR than the divergently transcribed *aphC *gene. Whether the 15 bp binding site suggested in *B. subtilis *is also specifically needed for binding in *Synechocystis*, hence, needs further investigation. Additional mechanisms involved in possible gene regulation by the PerR regulator in *Synechocystis *cannot be ruled out at present.

Given the possibilities mentioned in the previous paragraph, we investigated regulatory gene(s), which were coregulated with a large number of genes. These genes include *sll1771 *(coregulated with 113 genes), *rre12 *(64 genes), *slr2024 *(56 genes), *perR *(53 genes) and several others (see additional file 5). Some of these genes (*perR *and *rre12*) have been described in previous sections. The *sll1771 *gene encodes PphA, a PP2C-type phosphatase suggested to be involved in dephosphorylation of the PII protein in *Synechocystis *[39]. The PII protein has been suggested to be involved in sensing the C/N ratio in cyanobacteria [40]. Its activity is controlled by phosphorylation [39]. The large number of genes showing coregulation with the *pphA *gene was notable. Further analysis suggests a basis for correlation of the *pphA *expression along with its coregulated genes. As previously stated, cyanobacteria grown under photoautotrophic conditions acquire and fix carbon through the Calvin cycle using the energy generated by photosynthesis. The fixed carbon enters the glycolytic pathway and is utilized for nitrogen assimilation and for biosynthetic pathways. Assimilation of both C and N are energy intensive. Therefore, a tight correlation must exist on the expression patterns of genes involved in the various aspects of C and N metabolism. Because the PII protein is a global regulator of C/N assimilation and phosphorylation plays a key role in its activity, it is understandable that the expression patterns of so many genes coincide with that of the *pphA *gene. We also note that the expression pattern of the *glnB *gene, which encodes the PII protein, was correlated with the transcription of very few genes. These results suggest that regulation of C/N by the PII protein does not require significant changes in transcript levels of the *glnB *gene. Instead, a change in the transcript levels of the *pphA *gene is the key to regulating PII activity. We also found that expression patterns of a large number of ribosomal genes were correlated with the *nusG *(*sll1742*) gene. NusG is a regulator of gene expression known for complex and sometimes opposite effects on mRNA elongation [41].

### Identification of KEGG pathways responsive to perturbations

Our analysis of the CTR has led to the identification of key functional groups that are regulated in a concerted manner to allow cells to adapt under different perturbations. However, it is not clear how changes in a few major cellular processes affect others. Recent studies have shown that coordination between cellular pathways is the key to developing an appropriate adaptive response under perturbations [1,8,19,42]. During the analysis of transcriptome data sets, we noticed that some genes for a given cellular process were regulated in some data sets while a different set of genes for the same process were regulated in other data sets. It is highly possible that the regulation of a pathway is the same under diverse perturbations, although the changes are brought about by the regulation of different sets of genes in the pathway. Additionally, we observe that some KEGG pathways contain genes exhibiting negatively correlated expression dynamics. Such negative correlations of gene expression have been previously observed in other organisms, and may ultimately be related to interactions between various pathways [21,43]. Therefore, we next determined the regulation of KEGG pathways across perturbations.

**Figure 3:**
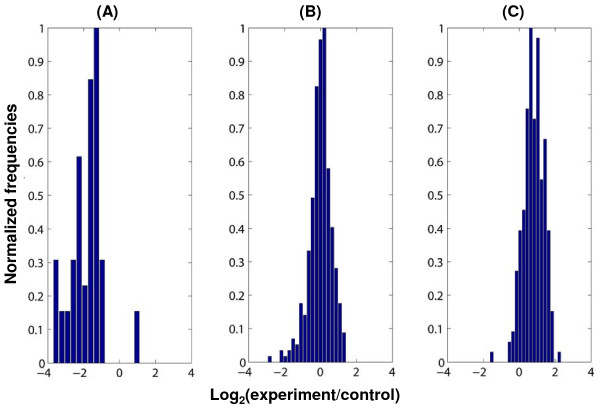
**Regulation of the functional category "Ribosome" determined from its associated genes**. Distribution of log_2 _(target/control) values of individual genes for the functional category "ribosome" are shown for (A) Singh_nitrogen_starvation', (B) 'KEGG_Hihara_hl_15 min' and (C) 'KEGG_Hihara_hl_15 h' (see additional file 6 for details). Based on the KS-test, "ribosomes" were either (A) downregulated, (B) not changed or (C) upregulated. Regulation of all KEGG pathways is provided in the additional file 6.

Based on Cyanobase annotations, there are currently 1582 genes (~45% (1582/3495)) with assigned functions in *Synechocystis*. Of these, 917 genes are associated with KEGG pathways, with some having multiple entries (1482 total entries). Accordingly, 60% of genes with known functions are currently associated with one of the 103 KEGG pathways listed for *Synechocystis*. In order to understand pathway level behaviors, expression levels of genes belonging to a pathway from individual data set were considered together and subsequently collapsed into a single value using the one-sample 'Kolmogorov-Smirnov (KS)' test [44] as described in the Methods section. Figure 3 shows an example of how the transcript levels of genes belonging to the component 'ribosome' are distributed among various experiments. The histogram in figure 3A corresponds to values observed under the 'Singh_nitrogen_starvation' experiment (additional file 6). Based on the KS test, this distribution was identified as being significantly different from a population with a mean of zero. Therefore, we assigned the value -1 indicating that the component ribosome was downregulated. Similarly, figure 3(B) and 3(C) show the corresponding histograms for the two experiments 'KEGG_Hihara_hl_15 min' and 'KEGG_Hihara_hl_15 h', respectively (additional file 6). Distributions in figure 3(B) did not meet the significance criterion and were assigned a value of 0, indicating the component ribosome was not differentially expressed. In contrast, the population in 3(C) had a positive mean, significantly different from zero and therefore, we assigned to it a value of +1, representing upregulation. Initially, we considered all KEGG pathways for which a gene has been assigned in *Synechocystis *(additional file 6). Further manual examination revealed that 51 KEGG pathways showed significant regulation across the different experimental conditions (additional file 7). Such an examination was necessary to remove bias introduced by transcriptome data sets generated from any single laboratory. Of the remaining KEGG pathways that were removed from further analysis, some pathways had either only a small number of *Synechocystis *genes in the pathway (less than 3 genes) or the number of differentially regulated genes was small.

### Coordination between KEGG pathways

**Figure 4:**
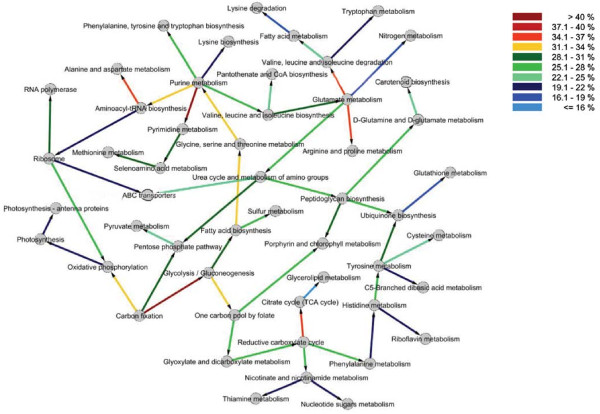
**Bayesian network for 51 KEGG pathways derived using the GES algorithm**. Based on regulation of their associated genes, 51 KEGG pathways were identified as significantly regulated across multiple perturbations. The Bayesian approach was used to generate the network. The color of the arrows represent the strength of the links, quantified using the True Link Strength Percent (see additional file 8).

To understand the coordination between various KEGG pathways under different perturbations, we generated a Bayesian network based on 51 selected KEGG pathways. A Bayesian network is a graphical model representing probabilistic relationships between different factors (in our case pathways) [45]. The nodes in the network correspond to random variables in the system while links between nodes represent probabilistic relationships. The compendium of large data sets made it possible to derive a realistic, systems level Bayesian network for *Synechocystis*. Such an approach has several desirable properties including a solid probabilistic background behind the algorithms, including an ability to combine data from different conditions, and an ability to make inferences of network changes under perturbations which can be tested in subsequent experiments. As expected, links in the network do not account for the same level of influence by parent nodes to the child node. We therefore quantified the link strengths by using the 'True Link Strength Percentage' as described in the Methods section. Figure 4 shows the resulting network for the selected 51 KEGG pathways. The various colors of the links represent the influence of the corresponding parent node on the child. The link strength percentages for the network varied between 15.8% and 45.8% (additional file 8). In addition, the link between two pathways does not always suggest positive correlation. It is quite possible that two pathways are linked together because the overall expression of the two pathways is consistently negatively correlated across various perturbations.

A powerful feature of the Bayesian network is making inferences on expected changes in the networks under different perturbations. This allows one to make predictions on possible modifications to the pathways so that a desired behavior is obtained from the system. There are several existing algorithms to perform inferences on Bayesian networks. Among these, the junction tree algorithm is one popular technique to get marginal probabilities of a Bayesian network given evidence(s) (see Methods for details). We have simulated several hypothetical perturbations using our network. We used three different scenarios: first, we downregulated the photosynthesis antenna system; second, we downregulated photosynthesis antenna system while simultaneously upregulating glycolysis, and finally, we downregulated both photosynthesis antenna system and glycolysis to understand how other KEGG pathways respond to these changes. Results of such conditional changes on the response of other pathways are shown and discussed in the additional file 9.

The analysis of network shows many expected as well as novel coordination(s) between various pathways. Moreover, it demonstrates how a specific effect on a particular pathway will impact the function of other cellular processes in *Synechocystis *(additional file 9). On a long term basis (if the perturbation persists), even an effect on a single pathway will eventually have a ripple effect on all other pathways. In essence, no pathway is an island. Since a significant amount of metabolic activity in *Synechocystis *is devoted to assimilation of C and N as well as protein synthesis, we have described in detail the coordination between KEGG pathways related to these metabolic activities, namely, glutamate metabolism, carbon fixation, and protein synthesis. The Bayesian network shows that glutamate metabolism in *Synechocystis *is probabilistically linked to the four other KEGG pathways involved in nitrogen assimilation *i.e*., nitrogen metabolism, arginine metabolism, urea cycle and metabolism of amino acids (Fig. 4). It is quite remarkable that a network derived from behavior of pathways based on transcriptional regulation shows close interdependence between various pathways involved in nitrogen metabolism. At the outset, the coordination between these pathways is significant because all these pathways are associated with the assimilation of nitrogen but utilizes different nitrogen sources such as nitrate, ammonium, urea, and arginine. It is also noteworthy that glutamate metabolism is the parent node connecting all other nitrogen pathways. This is significant, because all routes leading to the assimilation of N go through glutamate metabolism. The coordination between various pathways involved in nitrogen assimilation is also important given the fact that certain nitrogen assimilation pathways are energy intensive processes. It is therefore expected that the system would maximize resources under perturbation and utilize nitrogen sources that require minimal energy input. For example, the conversion of 1 mole of nitrate (present in the BG11 growth medium) to NH_4_^+ ^by GS-GOGAT cycle requires 8 moles of reduced ferredoxin [46]. Any perturbation that affects the photosynthetic process leading to lesser production of reduced ferredoxin would lead to an activation of pathways involved in transport and assimilation of other nitrogen sources. Indeed, we have recently found that illumination of *Synechocystis *with light that preferentially excites PSII leads to preferential utilization of an alternate nitrogen assimilation pathway over GS-GOGAT pathway [47].

A second node that is physiologically well understood involves carbon fixation. *Synechocystis*, like other photosynthetic organisms, utilizes energy produced from the photosynthetic light reactions to fix atmospheric CO_2_. It is expected that the rate of CO_2 _fixation will be tightly linked to the activity of photosynthetic electron transport chain. The Bayesian network shows that carbon fixation is probabilistically linked to three KEGG pathways, namely glycolysis, pentose phosphate pathway and oxidative phosphorylation (Fig. 4). It is interesting to note that the transcriptional regulation of genes involved in carbon fixation is not correlated with expression of photosynthetic genes but to expression of genes involved in oxidative phosphorylation. In fact, the link strength between carbon fixation and oxidative phosphorylation was one of the strongest in the network. Thus, our analysis shows the expected relationship between the rates of ATP production to carbon fixation. The interaction of carbon fixation with glycolysis and the pentose phosphate pathway is expected since ribulose-1, 5-bisphosphate, the primary acceptor for CO_2_, is regenerated by using the pentose phosphate pathway. Similarly, the product of carbon fixation, 3-phosphoglycerate, is channeled through glycolysis for either biosynthesis or glycogen accumulation. We also note that expression of glycolytic genes was not correlated with regulation of genes involved with the TCA cycle. It would be expected that flux changes in glycolysis will directly affect the flux through the TCA cycle and therefore expression patterns of genes encoding proteins involved in the TCA cycle should correlate with those of glycolytic genes. This lack of an interaction may appear surprising, but it is not an unlikely scenario considering the function of the TCA cycle in cyanobacteria. Unlike other organisms, cyanobacteria have an incomplete TCA cycle. It has been suggested that the TCA cycle in cyanobacteria is primarily utilized as a source for the production of precursors for N assimilation and biosynthesis [48]. Additionally, we have recently observed in a quantitative proteome study under varying environmental conditions that few substrates of TCA cycle can be regenerated from sources other than the glycolytic pathway (Wegener et al., unpublished results).

**Figure 5:**
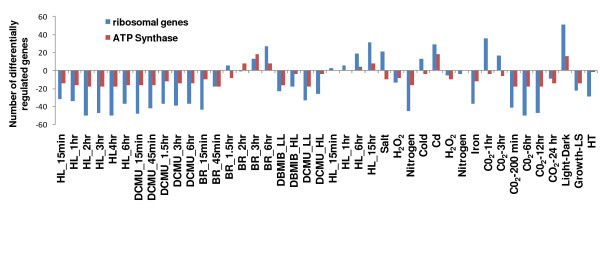
**Coregulation of ribosomal and ATP synthase genes**. Data sets with the significant number of differentially regulated genes corresponding to functional categories "ribosome" and "ATP Synthase" were used to determine coregulation. The details of experimental conditions and differential regulation of genes corresponding of these two functional categories are provided in additional files 1 and 2, respectively. The various abbreviations used are HL = high light; DCMU = 3-(3,4-Dichlorophenyl)-1,1-dimethylurea; DBMIB = 2,5-dibromo-3-methyl-6-isopropyl-*p*-benzoquinone; BR = blue and red light; H_2_O_2 _= hydrogen peroxide; Cd = cadmium; LS = linear-stationary; and HT = high temperature.

A third node of significant interest to the maintenance of homeostasis in *Synechocystis *is the function of the ribosome. Synthesis of the ribosome has been linked with the growth rate of organisms [49]. The Bayesian network in the present study shows that ribosome is probabilistically linked to oxidative phosphorylation, RNA polymerase and aminoacyl-tRNA biosynthesis (Fig. 4). While the coordination of the ribosome activity with RNA polymerase and aminoacyl-tRNA biosynthesis are expected, the link between the ribosome and oxidative phosphorylation is indeed fascinating. Ribosome function often depicts the status of cellular activity with faster growth leading to more ribosome synthesis and vice versa. Our analysis suggests that the expression of ribosomal gene is linked to ATP generation in *Synechocystis*. To look closer at the similarity of expression patterns between the ribosomal and ATP synthase genes, we compared the expression of genes belonging to these two processes under several perturbations (Fig. 5). We chose only those conditions where significant number of genes in these two processes was affected. Indeed, we found that the gene expression patterns in these two categories positively correlate to each other.

## Conclusion

In summary, large-scale transcriptomics data sets obtained under diverse perturbations on a cyanobacterium have been integrated. A compelling rationale for the importance of the present study is embedded in the sources of the data sets. The data sets were generated in multiple laboratories using different platforms with mostly undocumented physiological conditions of the cells. Despite these limitations, we have found that a large number of *Synechocystis *genes found on chromosome (~12%) are commonly regulated upon changes in the existing conditions. A majority of genes in this group belong to photosynthesis, energy metabolism, and translation, suggesting that these functions respond mostly to internal perturbation secondary to the primary stress. Additionally, genes involved in the two major energy producing pathways (photosynthesis and energy metabolism) and translation are mostly downregulated. These results provide evidence that a major adaptation strategy in *Synechocystis *is the regulation of genes in the anticipation of changes in growth during altered environmental conditions. We also identified coregulation of the CTR genes with regulatory genes which has provided insights into the regulatory networks in *Synechocystis*. We further utilized these large data sets to generate a Bayesian network based on KEGG pathways. This network has provided new insights on the coordination and interaction between various metabolic pathways in *Synechocystis*. Overall, our study has significantly increased the understanding of cellular strategies through the lens of transcriptomics in a photoautotroph. We have provided all data sets generated in this study, which will be a valuable resource to the entire cyanobacterial and plant research community for all gene expression related queries.

## Methods

### Collection of transcriptomics data sets

A total of 163 DNA microarray data sets were obtained from different sources. These include 83, 36 and 44 experiments from NCBI-GEO [50], KEGG expression [51] and from individual authors of the related literatures (additional file 1), respectively. These data sets were generated using either environmental perturbations (95) or genetic modifications (69). The environmental perturbations include changes in light quantity and quality, oxidative stress, metal stress, osmotic stress, high temperature, nutrients availability, treatments with DCMU and DBMIB. The additional file 1 provides the details of all environmental and genetic perturbations.

### Identification of gene- and pathway-levels behaviors

Raw data were processed using the robust version of LOWESS normalization [24]. The resulting values were used for subsequent computational analyses. For some data sets, we did not have access to the raw data and in those cases, the normalized data from the corresponding analyses were utilized.

To avoid the effects of local technical and biological variations, the differential behaviors of any individual gene were identified using statistical significance tests only. The level of shift in the sample means of replicates of individual gene expressions in each experiment was quantified using one sample KS-test [44]. In KS-test, we determine whether the observed log-ratio values were significantly different from a distribution with a zero mean. If the null hypothesis was rejected at a significance level of 5%, that gene was assigned +1 or -1 depending on whether the mean value was > 0 or < 0, representing an up and down regulation, respectively. If the null hypothesis could not be rejected, we assigned that gene a value 0, indicating that it was not differentially expressed. The entire results obtained from these analyses are available online at http://www.fibr.wustl.edu/synecho6803_database/.

To obtain function level behaviors, genes were grouped based on their KEGG metabolic pathway classifications. For any given KEGG pathway, expressions of individual genes were considered as population and pathways were assigned one of the three states (+1, 0 or -1) based on the KS-test. We further considered the number of genes in individual pathways and the percentage of differentially expressed conditions to select 51 pathways for further analysis.

### Gene regulatory network

A co-expression network was generated to identify the regulatory genes that are co-expressed with the CTR genes. Similarity between expression patterns of the CTR and regulatory genes was measured using the Hamming distance [52]. Expression patterns that agree more than 60% of times were identified and connections were drawn between corresponding genes. List of coregulated genes with the top 25 regulatory genes are available online at http://www.fibr.wustl.edu/synecho6803_database/.

### Derivation of associations between metabolic pathways using the Bayesian network approach

Selected 51 KEGG pathways were modeled using the Bayesian network approach. The Bayesian networks are graphical models representing the probabilistic relationships between different factors and have been extensively used in numerous fields [53]. The Bayesian Information Criteria (BIC) was used as performance measure in evaluating possible network structures. BIC is defined as,

where S is the structure of the network defining the nodes and the links between the nodes; θ_s _is the set of estimated parameters; D is the observed data given as an M × N matrix with N as the number of nodes (pathways) and M as the number of observations (experiments). The network structure was discovered using the Greedy Equivalence Search (GES) algorithm [54]. GES resulted in a network with a higher score compared to several other algorithms. More details of computational steps are provided in Elvitigala et al., [55].

### Determination of Regulatory Links Strength

To quantify the connection strengths of different links in the network, we used the 'True Link Strength Percentage', introduced by [56]. True link strength percentage measures the reduction of uncertainty on the state of the child node due to knowledge of the state of a parent node. It is computed as the ratio between reduction in entropy of the child node given the parent node and the original entropy of the child node.

### Network Inference for Different Experimental Conditions

We used the 'Junction Tree' algorithm to perform Network inference as previously described [45]. For this purpose, we fed the states of one or more pathways, which are expected to get significantly affected by the given experiment condition, as evidence to the network, and the changes in probabilities of other nodes are evaluated.

## Authors' contributions

AKS and HBP conceived and designed the study. TE designed the computational methods, performed the statistical analyses and wrote the methods section. AKS and JCC helped with the bioinformatics analyses. AKS and JCC analyzed and interpreted the results. MBP and BG contributed reagents/materials. AKS wrote the manuscript. All authors read and approved the final manuscript.

## Supplementary Material

Additional file 1 **Details of DNA microarray data sets generated in *Synechocystis *sp. PCC 6803 under various environmental and genetic perturbations**. This table lists details of transcriptome data sets used in this study. Click here for file

Additional file 2 **Fold change [log_2 _(treatment/control)] of genes across environmental and genetic perturbations**. This table lists the fold change under 68 experiments that were used for gene-level analyses. Click here for file

Additional file 3 **Discretized expression of genes across various perturbations**. This table gives the discretized value calculated from the additional file 2. Click here for file

Additional file 4 **Regulation of genes across perturbations**. This table gives the percentage of perturbations in which a given gene is differentially regulated. Click here for file

Additional file 5 **Correlation between known regulatory genes and core transcription response genes**. This table gives the distance value of 399 genes present in the CTR and 146 regulatory genes. Click here for file

Additional file 6 **Discretized value for each KEGG pathway calculated from 163 transcriptome data sets**. This table provides information on regulation of KEGG pathways in 163 data sets. Click here for file

Additional file 7 **Selected KEGG pathways which show significant differences across perturbations**. This table provides information on KEGG pathways selected to generate network using the Bayesian approach. Click here for file

Additional file 8 **True Link strength percent**. This table provides information on strength of connections between any two KEGG pathways. Click here for file

Additional file 9 **Network inference for different growth conditions**. This figure shows changes in probabilities of some selected pathways following changes in the probabilities of photosynthesis antennae proteins and glycolysis. Click here for file
