# Supplementary material for: Integrative analysis of large scale expression profiles reveals core transcriptional response and coordination between multiple cellular processes in a cyanobacterium
Source: BMC Syst Biol. 2010 Aug 2;4:105. doi: 10.1186/1752-0509-4-105 (PMC2924297; doi:10.1186/1752-0509-4-105)
Supplement: Additional file 9 — Network inference for different growth conditions. This figure shows changes in probabilities of some selected pathways following changes in the probabilities of photosynthesis antennae proteins and glycolysis. [file 1752-0509-4-105-S9.DOC]

**Additional file 9:** **Network inference following alteration of a specific pathway**. The Bayesian network was used to make inferences on how various pathways are modified following changes in the probabilities of ‘photosynthesis antennae proteins’ (PA) and ‘Glycolysis’ (G). We considered three conditions; (1) PA downregulated (PA-D), (2) both PA and G downregulated (PA-D-G-D) and (3) PA downregulated and G upregulated (PA-D-G-U). We used the junction tree algorithm to determine probabilities of selected 51 pathways used for the generation of the Bayesian network. We assigning appropriate states to PA and G (-1 for downregulation and +1 for upregulation), and changes in the nominal probabilities of selected cellular processes were observed. (A) and (B) demonstrate probabilities of pathways downregulated and upregulated, respectively. The results of three conditions have been compared with the original probabilities of KEGG pathways. As can be seen from (A) and (B), downregulation of PA (red bar) alone had minimal impact on selected pathways compared to original probabilities (blue bar). On the other hand, downregulation of both PA and G (green bar) had significant impact on carbon fixation, fatty acid biosynthesis and oxidative phosphorylation compared to the original probabilities. On the other hand, downregulation of PA and upregulation of G (purple bar) had significant impact on several cellular processes.

1. Probability of pathways being downregulated following changes in the probabilities of PA and G

1. Probability of pathways being upregulated following changes in the probabilities of PA and G
